# The Comparison of Antioxidant Performance, Immune Performance, IIS Activity and Gut Microbiota Composition between Queen and Worker Bees Revealed the Mechanism of Different Lifespan of Female Casts in the Honeybee

**DOI:** 10.3390/insects13090772

**Published:** 2022-08-26

**Authors:** Hongfang Wang, Li Lei, Wenfeng Chen, Xuepeng Chi, Kai Han, Ying Wang, Lanting Ma, Zhenguo Liu, Baohua Xu

**Affiliations:** College of Animal Science and Technology, Shandong Agricultural University, Tai’an 271018, China

**Keywords:** gut microbes, honeybee, lifespan, queen bees, worker bees, antioxidant, immune, IIS

## Abstract

**Simple Summary:**

Queen bees and worker bees are natural animal models for studying aging because queen bees live about 20 times longer than worker bees, despite both developing from fertilized eggs. Nutrition (intake of royal jelly or not) is a well-known external factor that causes the different lifespan between queens and workers. Exploring the nutrient-mediated molecular mechanism is of great significance to study the universal regularity of aging in animals. In this study, we systematically compared the differences of antioxidant, immune, IIS and gut microbiota composition between queens and workers. Of these, antioxidant, immune and IIS are the conserved mechanisms of aging. The gut microbiota are upstarts in regulating host health. We found that queens had stronger antioxidant capacity and lower immune pathway and IIS activity than workers. Queens also had supernal beneficial bacterium over workers. This study suggested that antioxidant, immune, IIS, and gut symbiotic bacteria all may contribute to the longevity of queens. This study provides more insights into revealing the mechanisms of queens’ longevity.

**Abstract:**

Queen bees and worker bees both develop from fertilized eggs, whereas queens live longer than workers. The mechanism of this phenomenon is worth exploring. Antioxidant capacity, immune and IIS are the conserved mechanisms of aging. The importance of gut bacteria for health prompted us to connect with bee aging. Therefore, the differences of antioxidant, immune, IIS and gut microflora between queen and worker bees were compared to find potential mechanisms of queens’ longevity. The results showed queens had stronger antioxidant capacity and lower immune pathway and IIS activity than workers. The higher expression level of *catalase* and *SOD1/2* in queens resulted in the stronger ROS scavenging ability, which leads to the lower ROS level and the reduced accumulation of oxidative damage products in queens. The lower *IMD* expression and higher antimicrobial peptides (AMPs) expressions in queens suggested that queens maintain lower immune pathway activity and stronger immune capacity than workers. Gut bacteria composition analysis indicated that queens had supernal Acetobacteraceae (notably *Commensalibacter and Bombella)*, *Lactobacillus* and *Bifidobacterium* over workers. In conclusion, antioxidant, immune, IIS, and gut symbiotic bacteria all contribute to the longevity of queens. This study provides more insights into revealing the mechanisms of queens’ longevity.

## 1. Introduction

Honeybees are highly eusocial insects, and their colonies consist of the queen, workers and drones. Queens and workers are both develop from fertilized eggs; however, they are different in reproduction, immunity, and other physiological functions [1,2,3]. Interestingly, the lifespan of queen bees is significantly different from that of worker bees, with queens living up to 10–20 times longer than workers [4,5]. Thus, honeybees have been studied as models of aging for a long time [6]. The changes in their lifespans are mostly caused by different diets. Throughout their lifetime, the queens primarily eat royal jelly, which is functionally analogous to mammalian breast milk and supplies complete nutrition, with antioxidative, antimicrobial, as well as immunoregulatory properties. In contrast, worker bees eat bee bread, the fermentation product of honey and pollen, for most of their short lives, except during the larval stage. Feeding royal jelly to worker bees leads to their longer lives [7]. It is of great significance in studying the molecular mechanism of the above phenomena.

The research advances in aging biology suggest that there may be three main molecular mechanisms explaining why queen bees live longer than worker bees. The first mechanism is the dynamic balance between oxidative damage and antioxidant capacity of the organism. The oxidative stress theory [8] has been widely accepted as a cause of organism senescence. Reactive oxygen species (ROS) is generated during the metabolism process, which attacks macromolecular substances (e.g., DNA, protein, and lipids) in cells and results in oxidative damage. The continuous accumulation of oxidative damage products leads to organism aging. In accordance with the above theory, it is speculated that there may be two reasons for the longevity of queen bees. Either queen bees produce less ROS, or queen bees have stronger ROS scavenging capacity than worker bees. An existing study on the larval stage showed that queen bee larvae produce higher ROS than worker bee larvae, but can be counterbalanced by the up-regulation of antioxidant genes [9]. Young queens (1d) have significantly higher expression of antioxidant genes than worker bees in most tissues, whereas older queens (1y) do not, so researchers have considered that queen bee longevity has evolved via mechanisms other than increased antioxidant gene expression [10]. Some studies in other social insects have disputed the idea that high expressions of antioxidant genes contributed to the longevity of queens. Research on termite queens supported this idea [11,12], whereas research on ant *Lasiusniger* has challenged this idea [13]. The second mechanism is immunosenescence. Immunosenescence refers to a common feature of organism senescence. IMD and Toll are the main signaling pathways that play a role in insect immune regulation [14,15]. IMD and Toll also respond to infection by regulating the expressions of antimicrobial peptides (AMPs) through the downstream transcription factors Relish and dorsal [16]. Infection of Gram-negative bacteria to young overwintering bees (November) significantly up-regulates the expression of Relish, while infection to old overwintering bees (January) leads to low expression of Relish, which reveals that aging decreases immune ability to fight infection [17]. Studies in fruit flies have also suggested that chronic immune activation can shorten lifespan [18,19]. Third, dietary restriction (DR) and inhibition of insulin/insulin-like growth factor signaling pathway (IIS) is a conserved mechanism for prolonging lifespan [20,21]. IIS is capable of interacting with vitellogenin (*vg*) [22], and the elevated *vg* can extend honeybee lifespan by protecting bees from oxidative stress [23]. Besides the above factors, intestinal bacteria take on a critical significance in host health.

Our latest study suggested that sucrose as overwintering food increased the Acetobacteraceae abundance in worker guts compared with honey and fructose syrup, and honeybee colonies with higher Acetobacteraceae abundances have less overwintering loss. The above results suggest that increasing Acetobacteraceae abundance in honeybee guts may prolong the lifespan of the bees [24]. Other studies have suggested that a queen bee’s gut bacteria, different from those of worker bees, may contribute to her longevity [25]. At the same time, gut microbes have been shown to be significantly correlated with IIS [26,27], the immune system [28], and the antioxidant system [29,30] in different species. Accordingly, the antioxidant performance, immune performance, IIS activity and gut microbial composition of the inchoate worker bees and queen bees were compared in this study to provide some basic data for exploring the molecular mechanism of the longevity of queen bees.

## 2. Materials and Methods

All experiments were conducted in the experimental apiary of Shandong Agricultural University (Tai’ an, Shandong Province, China). The beekeeping process follows the basic principles of honeybee welfare. 

### 2.1. Animals and Sampling

Three sister queen colonies (*Apis mellifera*) were used in the experiments of this study. The queen and worker bees were reared in accordance with the methods of Wang and Begna [31,32]. In brief, the spawning queens and an empty wax comb frame were confined to a cage for 12 h in a colony. Workers can pass through the cage freely, whereas the queens cannot. After the queens laid enough eggs, the frame was transferred to the upper successor box of the colony to incubate for 3 days. Some of the first-instar larvae were transferred from the worker cells to queen cell cups in a queen-rearing frame for further development. Seven days after emerging from the cells, five queen bees per colony, which had not finished mating but were sexually mature with a formed gut microflora community, were collected into 2-mL microcentrifuge tubes. The rest of the larvae remained in the worker cells and were allowed to develop into worker bees. Worker bees were marked with color after emerging from the cells. Seven-day-old worker bees were collected into 2-mL microcentrifuge tubes, of which the gut microflora community was stably formed. Under aseptic conditions, the guts of the 7-day-old virgin queens (*n* = 5/colony) and 7-day-old worker bees (*n* = 10/colony) were sampled to extract the gut microbial DNA [24]. Queen (*n* = 3/colony, *n* = 3/colony × 3 colony = 9) and worker bees (*n* = 4/colony, *n* = 3/colony × 3 colony = 12) were collected at 1, 3 and 5 days old for gene expressions and oxidative damage products assay, respectively.

### 2.2. Assay for Oxidative Damage Products

Honeybees were weighed and washed with ice-PBS to anaesthetize honeybees, and then they were homogenized in PBS (volume: weight = 9:1). Some protease inhibitors cocktail (0.2%, ab65621, abcam) was added into the PBS. The suspension was sonicated with an ultrasonic cell disrupter. Subsequently, the homogenates were centrifugated for 5 min at 5000× *g* to produce the supernatant. Half of the supernatant was tested for total protein using BCA Protein Assay Kit (Beyotime, Shanghai, China). The other half of the supernatant was used to detect ROS (Insect ROS ELISA Kit, Sino Best, Shanghai, China), Malonaldehyde (MDA, Insect MDA ELISA Kit, Sino Best, Shanghai, China), 4-hydroxynonenal acid (4-HNE, Insect 4-HNE ELISA Kit, Sino Best, Shanghai, China), protein carbonyl (PC, Insect PC ELISA Kit, Sino Best, Shanghai, China), as well as 8-hydroxydeoxyguanosine (8-OHDG, Insect 8-OHdG ELISA Kit, Sino Best, Shanghai, China) in accordance with the instructions. All indicators were corrected by being divided by the protein concentration.

### 2.3. RT-PCR

The total RNA of the respective bee was extracted with Trizol reagent (Invitrogen) and followed by reverse transcription to cDNA with the use of a reverse transcription kit (TaKaRa, Dalian, China). The mRNA expression levels of mRNA were examined through real-time PCR (7500 Real-time System, Applied Biosystems, Waltham, MA, USA) in a reaction volume of 20 μL with a SYBR PrimeScript RT-PCR kit (TaKaRa, Dalian, China). The following cycling conditions were employed, including 5 min at 95 °C (pre-incubation), 10 s at 95 °C (denaturation), 20 s at 60 °C (annealing), as well as 30 s at 72 °C (extension). Ten bees per cage were selected for qRT-PCR. Three technical replicates were performed for the respective sample. The β-actin gene (XM_017065464) served as the reference gene. Table 1 lists the sequences of the primers. Relative expression levels of genes were obtained using the 2^−ΔΔCt^ method.

### 2.4. Gut Microflora Genomic DNA Extraction and Sequencing

Microbial DNA was extracted with HiPure Stool DNA Kits (Magen, Guangzhou, China) in accordance with the manufacturer’s instructions. The DNA concentration, purity, and integrity were verified using a NanoDrop (Thermo, Waltham, MA, USA) (1.8 < A260/A280 < 2.0) and 1% agarose gels. The 16S rDNA V3–V4 regions of the ribosomal RNA genes were amplified through PCR using primers 341F: CCTACGGGNGGCWGCAG and 806R: GGACTACHVGGGTATCTAAT. Amplicons were extracted from 2% agarose gels and then purified with the AxyPrep DNA Gel Extraction Kit (Axygen Biosciences, Union City, CA, USA) according to the manufacturer’s instructions and quantified using the ABI Step One Plus Real-Time PCR System (Life Technologies, Foster City, CA, USA). The purified amplicons were pooled in equimolar quantities and then paired-end sequenced on an Illumina platform to generate 250-bp paired-end reads (PE250).

### 2.5. Bioinformatics Analysis of 16S rDNA Sequencing Data

The raw reads were filtered to remove reads containing over 10% unknown nucleotides and reads in which <50% of the bases exhibited a quality (Q-value) >20 in accordance with the following rules using FASTP (version 0.18.0) [33]. The paired-end clean reads were merged as the raw tags using FLASH (version 1.2.11) [34] with a minimum 10-bp overlap and a mismatch error rate of 2%. Noisy sequences of the raw tags were filtered through the QIIME pipeline (version 1.9.1) [35] to obtain high-quality clean tags. Clean tags were searched against the reference database (version r20110519, http://drive5.com/uchime/uchime_download.html, accessed on 8 December 2020) to perform reference-based chimera checking using the UCHIME algorithm [36]. All chimeric tags were removed. Afterward, the resulting effective tags were further analyzed. The effective tags were clustered and classified into operational taxonomic units (OTUs) at an identity threshold of 97% similarity with the use of UPARSE software (version 9.2.64) [37]. The representative OTU sequences were classified into taxonomic categories based on the SILVA database (version 132) [38].

### 2.6. Statistical Analysis and Software

Statistical comparisons between two measurements were conducted through unpaired two-tailed Student’s t-tests in SAS 9.1. The stacked bar plot of the community composition was visualized with the R project ggplot2 package (version 2.2.1). A heatmap of the species abundance was plotted using the pheatmap package (version 1.0.12) in R. Venn analysis was conducted using the R project VennDiagram package (version 1.6.16) to identify unique and common species. Biomarker features in the respective group were screened using LEfSe software (version 1.0) in R. A Bray–Curtis distance matrix was generated using the R project Vegan package (version 2.5.3). Multivariate statistical techniques, including principal coordinates analysis (PCoA) of the Bray–Curtis distances and pairwise analysis of similarities (ANOSIM) tests, were employed based on the R project Vegan package (version 2.5.3) to examine significant differences between the worker and queen gut microbial communities. *R* values in ANOSIM were adopted to detect the community overlap [39] (*R* > 0.75: well-separated; 0.50 < *R* ≤ 0.75: separated but overlapped; 0.25 < *R* ≤ 0.50: separated but strongly overlapped; *R* ≤ 0.25, barely separated). *p*-values suggested that there were significant differences between the groups (* *p* < 0.05, ** *p* < 0.01, and *** *p* < 0.001). The Kyoto Encyclopedia of Genes and Genomes pathway analysis of the OTUs was inferred using Tax4Fun (version 1.0). Functional differences between groups were obtained through Welch’s t-test in the R project Vegan package (version 2.5.3). Pearson correlation analysis between taxa and functions was conducted based on psych package (version 1.8.4). *p*-values were obtained through Fisher’s Z transformation.

## 3. Results

### 3.1. Antioxidant Performance of Queen Bees and Worker Bees

**Figure 1:**
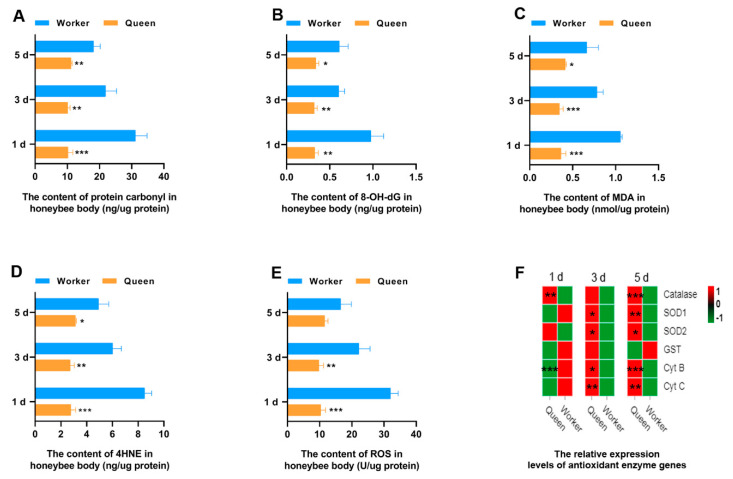
Antioxidant property. * represents *p* < 0.05, ** represents *p* < 0.01, *** represents *p* < 0.001, N_queen_ = 9, N_worker_ = 12.

Oxidative stress is considered as a biological aging mechanism, the core content of which is that ROS produced by body metabolism attacks large molecules (e.g., DNA, proteins, and lipids) within cells to produce the oxidative damage products. Aging is caused by the accumulation of oxidative damage products in the body. In the present study, the ROS and the oxidative damage products contents of DNA, proteins and lipids in queen and worker bees were measured (Figure 1). It was exciting to find that queen bees have significant lower levels of protein carbonyl (oxidative damage products of proteins), 8-OH deoxyguanosine (8-OH dG, oxidative damage products of DNAs), malondialdehyde (MDA), and 4-hydroxynonenal (4-HNE) (oxidative damage products of lipids) than worker bees at 1, 3 and 5 days old (Figure 1A–D). This result can be explained by the lower level of ROS of queen bees (Figure 1E). The ROS contents of queen bees at 1 day old (*p* = 0.000177) and 3 days old (*p* = 0.004435) were statistically lower than that of worker bees. In other words, the lower levels of ROS in queen bees result in less oxidative damage to its proteins, DNAs and lipids.

To explore the reasons for the lower ROS in queen bees than worker bees, the gene expressions of the key enzymes and mitochondrial proteins playing a role in the ROS elimination and respiratory function were determined through qRT-PCR. *SOD1* and *SOD2* play a critical role in the conversion of intracellular ROS to hydrogen peroxide. Next, hydrogen peroxide is broken down by catalase into H_2_O and oxygen. The results suggested that transcript levels of *SOD1*, *SOD2* and *catalase* were statistically different between queen bees and worker bees. *SOD1* (P_3d_ = 0.0205, P_5d_ = 0.0078) and *SOD2* (P_3d_ = 0.0486, P_5d_ = 0.0463) expression levels were significantly up-regulated in 3- and 5-day-old queen bees compared with worker bees (Figure 1F and Figure S1). The expression levels of *catalase* (Figure 1F) in queen bees were significantly higher than that in worker bees at 1 day old (*p* = 0.001183) and 5 days old (*p* = 0.000070). Cyt B and Cyt C are the mitochondrial proteins involved in the respiratory process; Cyt B was found to be lower in 1-day-old queen bees than 1-day-old worker bees (P_1d_ = 0.000720). However, the expression levels of *Cyt B* (P_3d_ = 0.01802; P_5d_ = 0.000140) and *Cyt C* (P_3d_ = 0.002951; P_5d_ = 0.002593) in queen bees were significantly higher than those of worker bees with the increase of age. Glutathione-S-transferase (GST), a key enzyme in glutathione binding reaction, plays an important role in the elimination of ROS. It was found that the *GST* expression of queen bees was not superior to that of worker bees.

### 3.2. The Immune Performance of Queen Bees and Worker Bees

**Figure 2:**
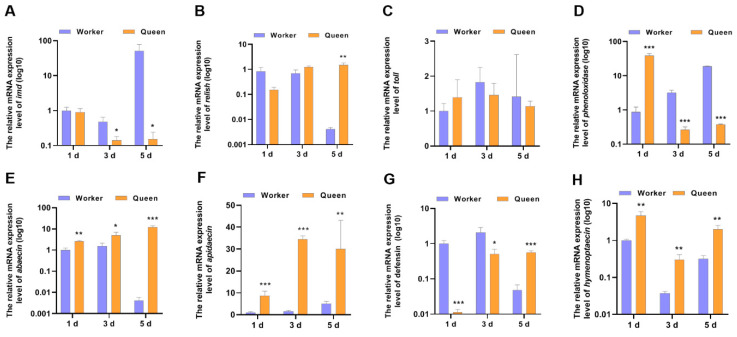
The mRNA expression levels of the genes involved in immune. * represents *p* < 0.05, ** represents *p* < 0.01, *** represents *p* < 0.001, N_queen_ = 9, N_worker_ = 12.

The declined immune capacity is a common feature of animal senescence [40]. Aging is correlated with changes in immunity. So, IMD pathway, AMP and other immune-related genes, as indicators to evaluate the immune performance of honeybees, were determined (Figure 2). The mRNA expression level of IMD in 3-day-old queen bees (*p* = 0.029202) and 5-day-old queen bees (*p* = 0.039897) significantly decreased compared with worker bees. However, the mRNA expression of *Relish,* downstream transcriptional factor of imd, did not decrease in queens. In contrast, *Relish* mRNA expression of queen bees was higher than worker bees at 5 days old (*p* = 0.001252). As the downstream target genes of transcriptional factor *relish*, AMPs expression trends were not consistent with *relish*. Most AMPs mRNA expressions (excluding *defensin*) in queen bees were up-regulated at 1, 3 and 5 days old. Only *defensin* mRNA levels was lower in queens than in workers at 1 day old and 3 days old. These indicated that the expressions of AMPs genes in bees were also regulated by another transcriptional regulatory manner. Another immune-landmark gene, phenoloxidase, was also detected. At the age of 1 day, the mRNA level of phenoloxidase in queen bees was increased to ~2-fold that of worker bees’ phenoloxidase mRNA level (*p* = 0.000045). Nevertheless, at the ages of 3 and 5 days, the *phenoloxidase* mRNA level of queen bees decreased compared with that of worker bees.

### 3.3. The Activity of Insulin/Insulin-Like Growth Factor Signaling Pathway of Queen Bees and Worker Bees

**Figure 3:**
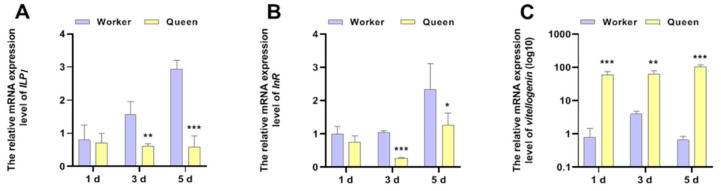
The mRNA expressions of *ILP1, InR* and *vitellogenin.* * represents *p* < 0.05, ** represents *p* < 0.01, *** represents *p* < 0.001, N_queen_ = 9, N_worker_ = 12.

Except for the function of being a pathway response to nutrition, the function of IIS in senescence regulation is conserved from nematodes to higher animals. *vg*, a signature molecular of organismal nutrition reserve, has been considered to be significantly correlated with the longevity of honeybees. The mRNA expressions of *ILP1*, *InR* and *vg* were shown in Figure 3. The results displayed that the *ILP1* (P_3d_ = 0.003971, P_5d_ = 0.000696) and *InR* (P_3d_ = 0.000033, P_5d_ = 0.010141) mRNA expression levels of queen bees were lower than that of worker bees at 3 days old and 5 days old, thus revealing that queen bees exhibited lower activity of IIS. However, the phenomenon was not significant in newly emerged honeybees (1 day of age). The caste-expression pattern of *vg* supported the longevity phenotype of queen bees more than worker bees. At the age of 1 day (*p* = 0.000498), 3 days (*p* = 0.002875) and 5 days (*p* = 0.000106), *vg* was overexpressed in queen bees compared with worker bees.

### 3.4. Queen Bees and Worker Bees Had Different Gut Microbial Profiles

Gut microflora was proved to regulate host immune and nutrition metabolism, two vital mechanisms of aging in animals. Thus, it is inferred that gut microflora may affect host longevity by interacting with host immune and nutrition metabolism. As inspired by this inference, we explored the different gut bacterial community compositions between queen bees and worker bees.

There were differences in the gut microflora composition between workers and queens (Figure S2). Venn diagrams were constructed to analyze the common and unique OTUs between worker bees and queen bees. The worker and queen bees’ guts contained 339 common OTUs (50.67%); the worker guts contained 266 unique OTUs (43%), and the queen guts contained 346 unique OTUs (50.51%) (Figure S3).

**Figure 4:**
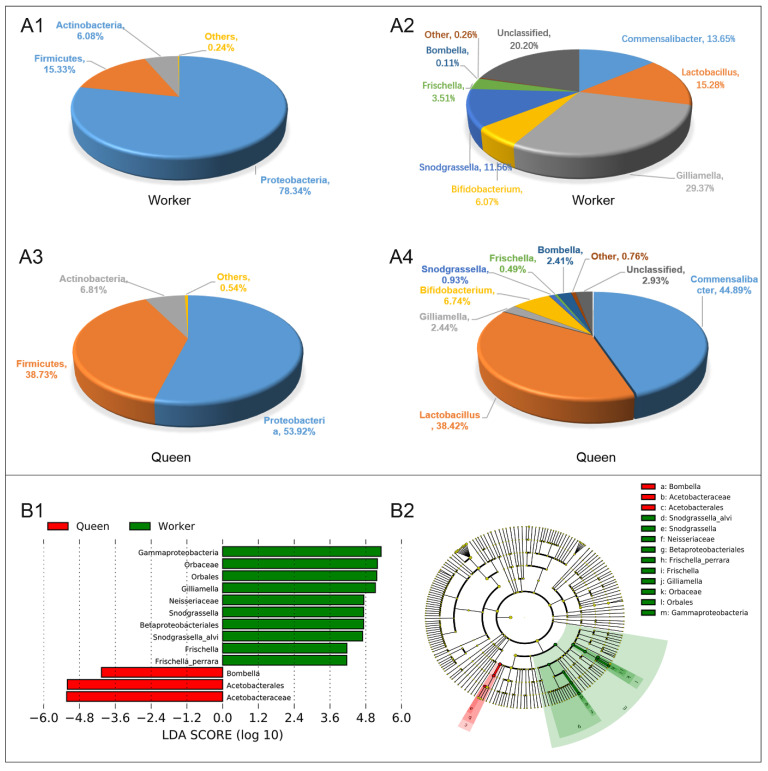
Gut microbial abundance and taxonomic distribution of the workers and queens. (**A1**). Gut microbial composition and relative abundance of worker bee at phylum level; (**A2**). Gut microbial composition and relative abundance of worker bee at genus level. (**A3**). Gut microbial composition and relative abundance of queen bee at phylum level; (**A4**). Gut microbial composition and relative abundance of queen bee at genus level; (**B1**). LEfSe analysis (taxa with LDA scores > 4). (**B2**). Cladogram of the biomarker main taxa of the microbiotas based on LEfSe analysis. Green represents significantly different taxa with the highest relative abundances in worker guts; red represents significantly different taxa, with their highest relative abundances in the queen gut.

Queen and worker bees have similar taxonomic types of dominant gut microflora at the phylum level, including Proteobacteria (78.34% for workers, 53.92% for queens), Firmicutes (15.33% for workers, 38.73% for queens) and Actinobacteria (6.08% for workers, 6.81% for queens) (Figure 4A1,A3). However, the guts of worker and queen bees were different in the dominant genera. The dominant microflora in worker guts at the level of genus were *Gilliamella* (29.37%), *Lactobacillus* (15.28%), *Commensalibacter* (13.65%), *Snodgrassella* (11.56%), *Bifidobacterium* (6.07%) and *Frischella* (3.51%) (Figure 4A2). The dominant microflora in queen guts at the level of genus comprised *Commensalibacter* (44.89%), *Lactobacillus* (38.42%), *Bifidobacterium* (6.74%), *Gilliamella* (2.44%) and *Bombella* (2.41%) (Figure 4A4). Compared with the worker bees, queens had higher abundances of Alphaproteobacteria Acetobacterales Acetobacteraceae (*Commensalibacter and Bombella*), Firmicutes Bacilli Lactobacillales Lactobacillaceae (*Lactobacillus*), as well as Actinobacteria Bifidobacteriales Bifidobacteriaceae (*Bifidobacterium*). LEfSe analysis (Linear Discriminant Analysis, LDA > 4) was performed to determine the caste-biased biomarker taxa. Queen-biased biomarker taxa included *Bombella intestini*, and the worker-biased biomarker taxa consisted of *S. alvi*, *F. perrara* and *G. apicola* (Figure 4B1,B2).

## 4. Discussion

The initial cause of the difference in the lifespans between queen and worker bees is nutrition. Animal aging and longevity are regulated by some conservative signaling pathways including immune (IMD), antioxidant and nutrition response pathway (IIS). Our study indicated that there are differences in immune performance, antioxidant ability and IIS activity between queen and worker bees. A question is raised whether there is a bridge between nutrition and signaling pathways; if so, the bridge may be gut bacteria since gut microbiota are the first “friends” nutrients meet when they enter the organism. Gut microbiota, as a vital regulatory factor of host nutrition and immunity, has also become a new luxury in aging biology. Next, we want to discuss the potential roles and interactions of immune, antioxidant, IIS and gut microbiota in the different adult longevity of honeybees.

The oxidative stress theory [8] is a widely accepted cause of organism senescence. ROS is generated during the metabolism process, which attacks macromolecular substances (e.g., DNA, protein, and lipids) in cells and causes oxidative damage. The continuous accumulation of oxidative damage products in the body leads to organism aging. The prediction from this theory is that long-lived queen bees may have lower rates of ROS production, or long-lived queen bees may have more powerful ability of eliminating ROS. To answer this question, antioxidant genes involved in eliminating ROS (*SOD1*, *SOD2, catalase*, *GST*) and mitochondrial proteins involved in respiratory (*Cyt B* and *Cyt C*) were assayed. The results suggested that queen bees did have lower lipids, protein and DNA oxidative products and ROS. Higher expression levels of *Cyt B* and *Cyt C* in queen bees suggested that queen bees have stronger oxidative respiration than worker bees, which means queen bees do not reduce ROS production compared with worker bees. It was found that queen bees showed superiority in the expressions of antioxidant genes over worker bees. For instance, the expressions of *SOD1/2* and *catalase* in queen bees were superior to worker bees. This means that queen bees are more efficient at removing ROS. That may be one reason for its longevity. What up-regulates antioxidant enzyme gene in queen bees? It was found that queen bee larvae were rich in coenzyme Q10 [41], and feeding coenzyme Q10 to worker bees could improve antioxidant enzymes [42]. We speculated that the higher coenzyme Q10 in queen bee larvae may come from two sources, one from queen bees’ food, royal jelly, and the other from the special gut flora of queen bees that is different from worker bees. The analysis of coenzyme Q10 content in lyophilized royal jelly and beebread (worker bee’s food) indicated that coenzyme Q10 content in royal jelly (8 ± 0.2 µg/g) was not higher than that in beebread (11.5 ± 0.3 µg/g) [43]. Thus, queen bee special gut microbiota is more likely to be causal for the higher coenzyme Q10 in queen bees. In this study, a queen bee special biomarker microbiota, *B. intestini*, was salvaged through LEfSe analysis. *B. intestini* was first isolated from bumblebee crops [44], and is part of a clade of acetic acid bacteria (a group within the family Acetobacteraceae). To date, *B. intestini* from honeybees has not been found. However, *B. apis*, another member of the genus *Bombella* with 98% sequence similarity to *B. intestini*, was detected from honeybee midguts [45]. However, little is known about the role of genus *Bombella* in honeybee fitness. Whole-genome sequencing of *B. intestini* and *B. apis* has suggested that the major ubiquinone of *B. intestini* and *B. apis* is Q10 [46,47]. The above scientific evidence supported a logical hypothesis that queen bee-biased biomarker bacteria *B. intestini* makes a positive contribution to the longevity of queen bees. Indeed, realistic experiments should be further performed to verify this hypothesis.

Immunosenescence is a common feature of animal senescence [40]. IMD and Toll were the main immunomodulatory pathway in insects [15,48]. IMD and Toll can also regulate the expression of *AMPs* by downstream transcription factors *Relish* and *dorsal* [16,49]. IMD and Toll have been found to be up-regulated in aging *drosophila* [50]. Sustained up-regulation of *imd* indicates immune activation, and the overactivated immune response, which is a drain to the organism, have been proved to shorten the lifespan of fruit flies [51]. The results of this study showed that *IMD* in queen bees was suppressed compared with worker bees, which means that the queen bees have less immune expenditure. This is concordant with the fact that queen bees live longer than worker bees. Curiously, IMD-downstream transcription factor, *Relish*, was not Wdown-regulated in queen bees. Inversely, the expression of *Relish* in 5-day-old queen bees was higher than that in worker bees. This may lead directly to up-regulation of most downstream *AMPs* genes in queen bees at 5 days. The up-regulated *AMPs* mRNA expressions (excluding defensin) in 1-day and 3-day-old queen bees indicated that the expressions of *AMPs* genes in bees were also regulated by other transcriptional regulatory manners. The contradict of *IMD* and *Relish* expressions is a phenomenon worth discussing. IMD was mainly activated by Gram-negative bacteria including pathogenic bacteria [52,53] and normal symbiotic bacteria [49]. Among the gut symbiotic bacteria of honeybee, only *G. apicola* and *S. alvi,* the worker’s dominant taxa, are Gram-negative bacteria [54]. The dominant microflora in the queen guts (*Commensalibacter*, *Lactobacillus* and *Bifidobacterium*) are primarily Gram-positive bacteria [55,56], which may be one of the reasons why the *imd* expression level of queen bees is lower than that of worker bees.

A significant difference between queens and workers is their nutrition, which may contribute to their differential longevity. The queens primarily eat royal jelly throughout their lifetime. In contrast, worker bees eat honeybee bread, the fermentation product of honey and pollen, for most of their short lives, except during the larval stage [57]. Feeding royal jelly to worker bees enables them to live longer [7]. Previous research on aging has suggested that the regulatory pathways of nutrition are significantly correlated with senescence. Inhibited IIS has been proved as a conserved molecular mechanism of extending lifespan [20,21,58]. The previous study proved that only *ILP1* of the two *AmILPs* can respond to diet [59], so we investigated *ILP1* expression in queen and worker bees. *vg* interacts with components of the insulin signaling pathway, which has been increasingly considered as a vital regulator of honeybee lifespan [22]. The results of this study suggested that IIS activity of queen bees was suppressed, obviously compared with worker bees. The above results are consistent with the findings of Corona [22]. It is noteworthy that studies on *Drosophila melanogaster* have found that high nutrient levels up-regulate IIS, thus shortening life expectancy [60]. In honeybees, however, IIS of queen bees fed with higher nutrient levels than worker bees is down-regulated. What makes honeybees different? The gut microbes of *Drosophila* and *Apis mellifera* can interact with host IIS [26,27]. The different gut microbiota composition between queen bees and worker bees may exert different domino effects on IIS. *vg* expression in germ-free worker bees is down-regulated through the mono-inoculation with *S. alvi* and then infected with a trypanosomatid parasite under hive conditions. As *vg* is found to extend lifespan in honeybees [23], the above supported observations in this study that long-lived queen bees possessed lower *S. alvi* abundance and higher *vg* expression levels than short-lived worker bees. The above also suggested a role of the gut microbiota in influencing bee lifespan through interacting with IIS and *vg*.

In eusocial insects, there were various phenotypes between queens and worker bees, which consisted of morphology, genitalia and longevity. Studies on the differences in longevity between castes are valuable because individual longevity is often significantly correlated with physical fitness. Recent studies on how microbial communities in bee guts play a role in pathogen protection and nutrition metabolism have aroused attention to the effects of the microbiota on bee fitness. Thus, the differences in the gut microbial communities of the queen and worker bees and whether they would contribute to differences in their lifespans were examined. Existing studies on the gut microbial communities of queens and worker bees suggest that queens lack the stable core microbiotas that are correlated with the workers, though the above studies have employed queens of different ages and reproductive stages (i.e., 4–6-month-old, 16–18-month-old, 4-day-old virgin, 14-day-old spawning and 7-day-old virgin queens) [25,61,62]. This study on caste-biased microbiotas in *Apis mellifera* reconfirmed the significant differences between castes. The same phenomenon was identified in termites, which were also social insects [63,64]. Different gut microbial communities between infertile and royal termite castes have been suggested to be correlated with caste-specific diets and lifestyles [64], and the same is true for honeybees. Compared with the workers’ gut microbial data reported by Kwong and Moran [56], Anderson et al. [25] have found that the queen-specific microflora of *Apis mellifera ligustica* consists of *Parasaccharibacter apium* (Alpha-2.2) and *Lactobacillus kunkeei* (Firm-5); worker-specific microflora comprises *Bartonella apis*, *F. perrara*, *S. alvi*, as well as *G. apicola*, and shared core microflora contains *Lactobacillus Firm-4*, *Lactobacillus Firm-5*, *Bifidobacterium asteroids*, as well as Acetobacteraceae Alpha 2.1. As revealed by the results of this study, the queen-biased biomarker taxon was *B. intestini* (Alpha 2.2), the worker-biased biomarker taxa consisted of *S. alvi*, *F. perrara*, and *G. apicola*, and queens achieved higher abundances of *Commensalibacter*, *Lactobacillus*, and *Bifidobacterium* than worker bees. However, *B. intestini* was not the most abundant bacterium in the queens, but the fifth most abundant, with *Commensalibacter* (44.89%; Alpha 2.1), *Lactobacillus* (38.42%), *Bifidobacterium* (6.74%), and *Gilliamella* (2.44%) ranking before it (2.41%). As reported by Powell et al., Acetobacteraceae (Alpha-2.1 and Alpha-2.2) and *Lactobacillus Firm-5* dominate queen guts [62]. Kapheim reported similar results that the top four most abundant bacteria include *P. apium*, Alpha-2.1, *Lactobacillus Firm-4*, and *Lactobacillus Firm-5* [61]. The above studies all suggest that queen guts have more abundant Acetobacteraceae, especially Alpha-2.2, than worker guts, which is probably correlated with the honeybee diets. Existing research has concluded that Acetobacteraceae Alpha 2.2 is prolific in the crops, hypopharyngeal glands of nurse bee, royal jelly, and larva fed on royal jelly, whereas it is negligible in the nurse and forager midguts and guts [65,66,67]. Some niches are characterized by the availability of royal jelly, which is the main food of queen bees and is different from bee bread and honey eaten by the workers. Thus, the guts of queen bees are a niche accessible to royal jelly, thus suggesting that royal jelly is likely to facilitate Acetobacteraceae Alpha 2.2 proliferation. The widespread distribution of Alpha 2.2 in numerous niches of honeybees reveals some specialized biological functions in the hosts. Our latest study has suggested that overwintering honeybee colonies with higher abundances of Acetobacteraceae exhibits a lower rate of overwintering loss [24], which reveals that Acetobacteraceae plays a positive role in honeybee fitness. One Alpha 2.2 isolate has been found to increase honeybee larval survival in vitro [67]. Acetobacteraceae are symbionts of various insects. Existing studies on *Anopheles* and *Drosophila* indicate that Acetobacteraceae provides nutrition to the host [68], contributes to host growth and development [27,69,70], and modulates host immunity [49]. Accordingly, this study reveals that the abundant Acetobacteraceae, including *Commensalibacter* and *Bombella*, in the guts of queen bees, may be another vital beneficial bacterium enabling queen bees to live healthier and longer than worker bees, except for the well-known beneficial symbiotic bacteria *Lactobacillus* and *Bifidobacterium.*

## Figures and Tables

**Table 1:** Primer sequence for RT-PCR.

Genes	Primer Sequences
*ILP1*	F′-GCTCAGGCTGTGCTCGAAAAGT
	R′-CGTTGTATCCACGACCCTTGC
*InR1*	F′-ACGGGATGGCCTACTTGGAG
	R′-GGAAACCATGCAATTCCTCG
*vg*	F′-AGTTCCGACCGACGACGA
	R′-TTCCCTCCCACGGAGTCC
*Catalase*	F′-GGCGGCTGAATTAAGTGCTA
	R′-TTGCGTTGTGTTGGAGTCAT
*GST*	F′-CAATTTGATGAACGGGGAAC
	R′-GCCGTACCGATGTTTTCGTA
*Cyt B*	F′-CCAACTCATATTAAACCTGAATG
	R′-CCGATTACACCTCCTAATTTATT
*Cyt C*	F′-CACAAAGTAGGACCTAATCTTTATGGAGTA
	R′-TCCTTTATTCGCATCTGTGTAGCT
*SOD1*	F′-GTCGTTCCGTGTAGTCGAGAA
	R’-TCCTTTGACTTCACCCTGAAGA
*SOD2*	F′-GGTGGTGGTCATTTGAATCATTC
	R′-AAGAAGTGCAGCGTCTGGTTTAC
*IMD*	F′-TGTTAACGACCGATGCAAAA
	R’-CATCGCTCTTTTCGGATGTT
*Relish*	F′-GATGCAGAAGATGAAAAAGCAG
	R′-TGAACACATTTCGTTTGTTGTTT
*Toll*	F′-TAGAGTGGCGCATTGTCAAG
	R′-ATCGCAATTTGTCCCAAAAC
*Phenoloxidase*	F′-AGATGGCATGCATTTGTTGA
	R′-CCACGCTCGTCTTCTTTAGG
*Hymenoptaecin*	F′-CTCTTCTGTGCCGTTGCATA
	R′-GCGTCTCCTGTCATTCCATT
*Defensin*	F′-TGCGCTGCTAACTGTCTCAG
	R′-AATGGCACTTAACCGAAACG
*Abaecin*	F′-CAGCATTCGCATACGTACCA
	R′-GACCAGGAAACGTTGGAAAC
*Apidaecin*	F′-TTTTGCCTTAGCAATTCTTGTTG
	R′-GTAGGTCGAGTAGGCGGATCT
*β-actin*	F′-CCGTGATTTGACTGACTACCT
	R′-AGTTGCCATTTCCTGTTC

## Data Availability

The dataset supporting the conclusions of this article is available in the NCBI Sequence Read Archive database (Accession Number: SUB8289109).

## Supplementary Materials

The following are available online at https://www.mdpi.com/article/10.3390/insects13090772/s1, Figure S1: The mRNA expression levels of the genes involved in antioxidation. * represents *p* < 0.05, ** represents *p* < 0.01, *** represents *p* < 0.001, Nqueen = 9, Nworker = 12, Figure S2: Diversity differences in the gut microflora between workers and queens. A. Principal coordinate analysis (PCoA) plot based on Bray-Curtis similarity for the worker and queen samples. B. Analysis of similarities (ANOSIM) testing based on unweighted UniFrac was used to evaluate the intergroup (workers and queens) and intragroup (workers or queens) distances. R, interpretation degree of the different groups in the sample differences. C. Sample distance analysis based on the Bray-Curtis distance index. Larger indices indicate greater distances between samples, Figure S3: Numbers of OTUs in the worker and queen guts.
