# Supplementary material for: The Comparison of Antioxidant Performance, Immune Performance, IIS Activity and Gut Microbiota Composition between Queen and Worker Bees Revealed the Mechanism of Different Lifespan of Female Casts in the Honeybee
Source: Insects. 2022 Aug 26;13(9):772. doi: 10.3390/insects13090772 (PMC9506344; doi:10.3390/insects13090772)
Supplement: Supplementary file 1 [file insects-13-00772-s001.zip › insects-1788437-supplementary.pdf]

Supplementary Materials

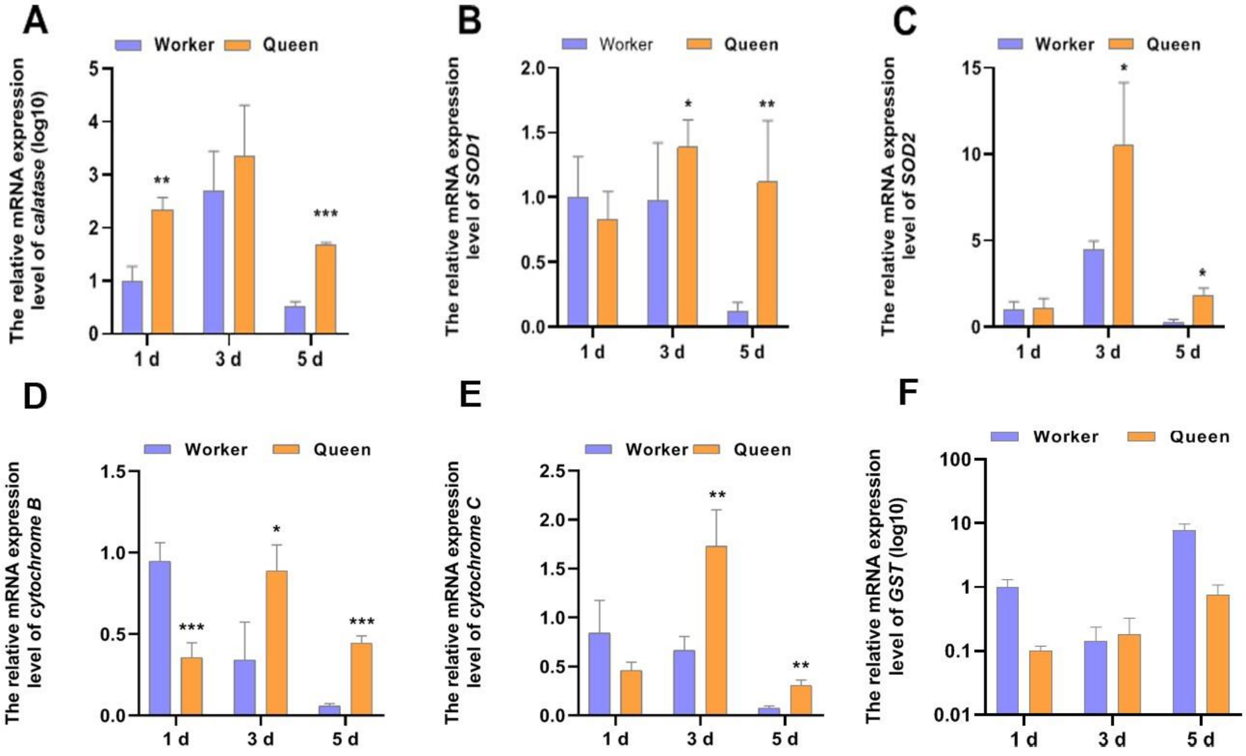

Figure S1. The mRNA expression levels of the genes involved in antioxidation. * represents $p < 0.05$, **represents $p < 0.01$, *** represents $p < 0.001$, Nqueen= 9, Nworker=12.

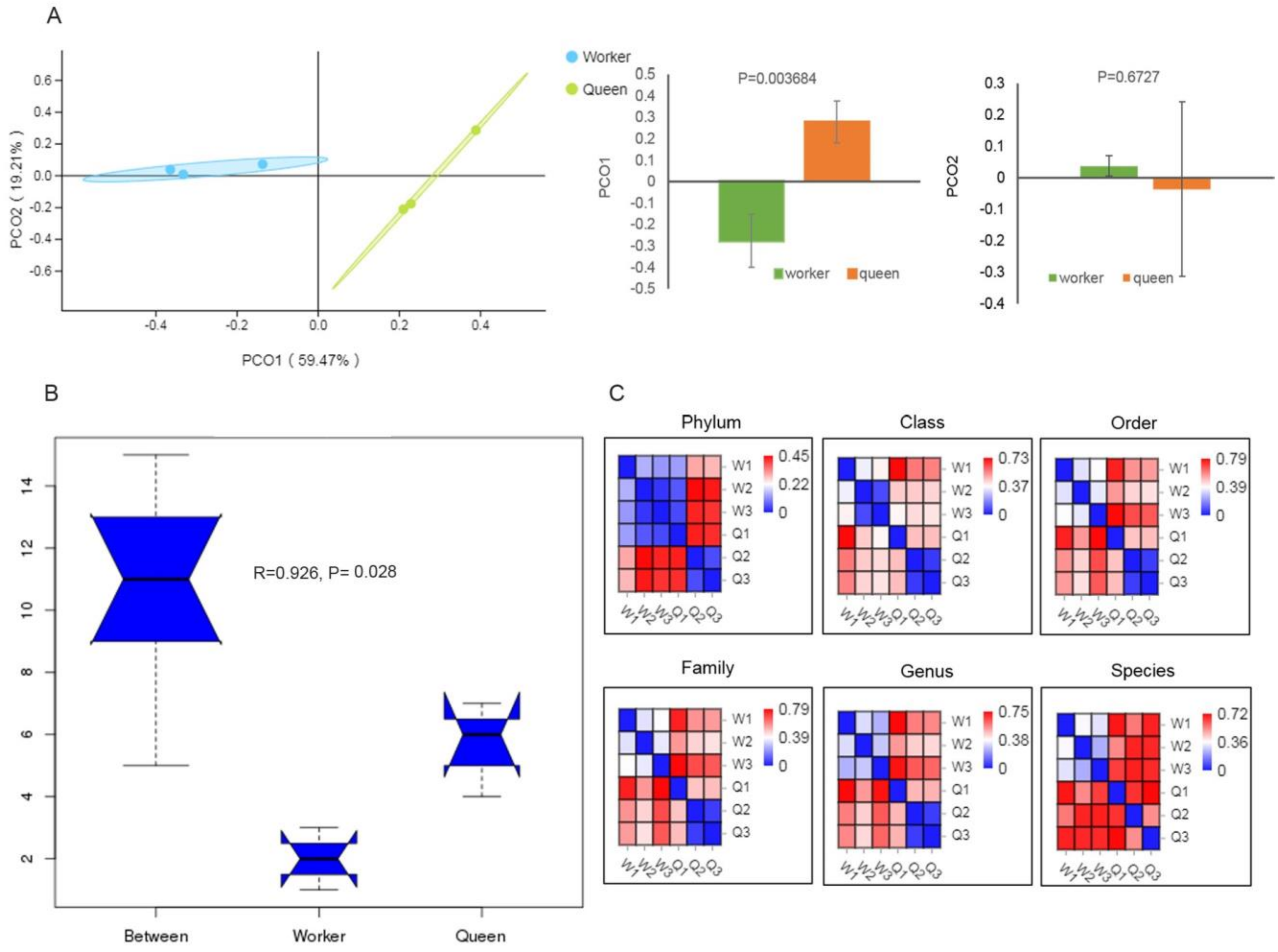

Figure S2. Diversity differences in the gut microflora between workers and queens. A. Principal coordinate analysis (PCoA) plot based on Bray-Curtis similarity for the worker and queen samples. B. Analysis of similarities (ANOSIM) testing based on unweighted UniFrac was used to evaluate the intergroup (workers and queens) and intragroup (workers or queens) distances. R, interpretation degree of the different groups in the sample differences. C. Sample distance analysis based on the Bray-Curtis distance index. Larger indices indicate greater distances between samples.

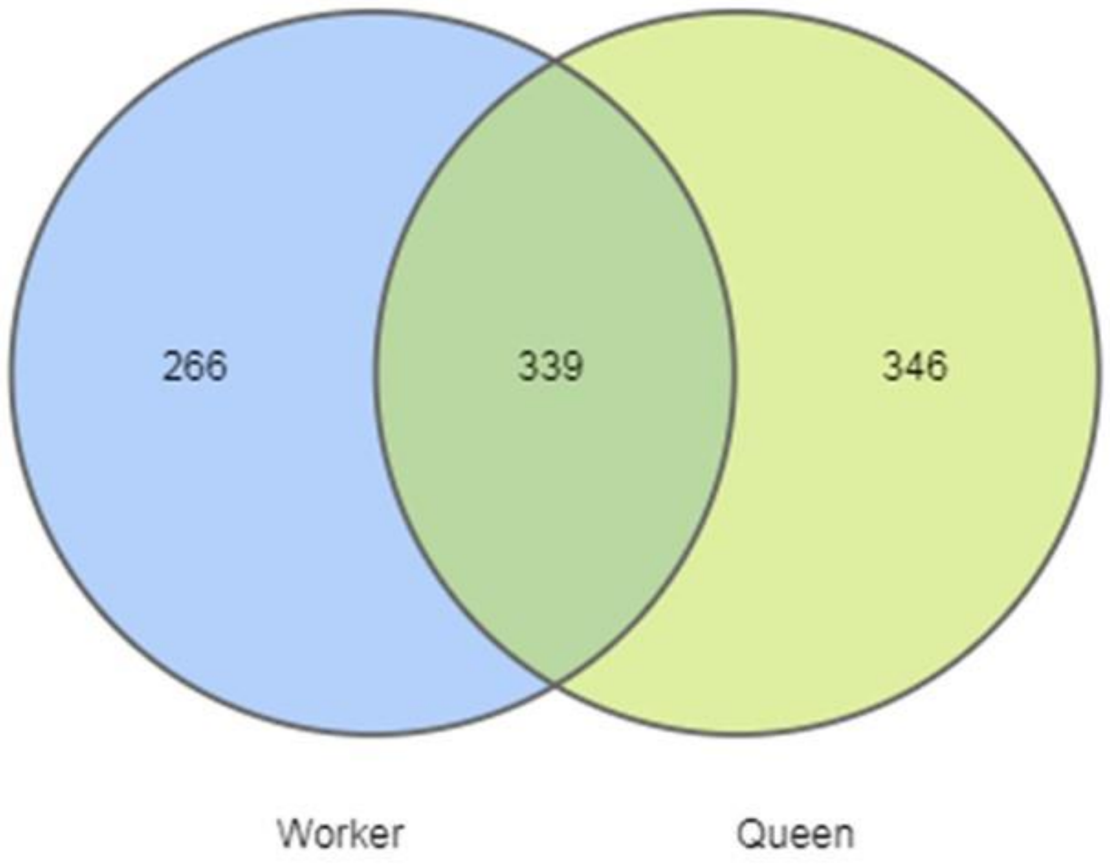

Figure S3. Numbers of OTUs in the worker and queen guts.
